# Effect of cryoprecipitate on an increase in fibrinogen level in patients with excessive intraoperative blood loss: a single-center retrospective study

**DOI:** 10.1186/s40981-022-00516-5

**Published:** 2022-04-05

**Authors:** Satoshi Kouroki, Toyoaki Maruta, Isao Tsuneyoshi

**Affiliations:** 1 Department of Anesthesiology, Nichinan Prefectural Hospital, Kiyama 1-9-5, Nichinan, Miyazaki, 887-0013 Japan; 2 Department of Anesthesiology, University of Miyazaki Hospital, Kihara 5200, Kiyotake-cho, Miyazaki, 889-1692 Japan

**Keywords:** Fibrinogen, Cryoprecipitate, Intraoperative massive bleeding

## Abstract

**Background:**

Cryoprecipitate, which contains fibrinogen and factor VIII in large quantities, is concentrated from fresh frozen plasma, and it has hemostatic effects in severe bleeding. We retrospectively examined the effects of cryoprecipitate on the increase in fibrinogen levels in patients with excessive intraoperative blood loss.

**Methods:**

Ninety-seven patients who were administered cryoprecipitate during surgery between June 2014 and May 2019 were enrolled in our study and categorized according to the volume of intraoperative blood loss as follows: group A, 2000–5000 mL; group B, 5000–10,000 mL; group C, > 10,000 mL. Data were extracted from electronic medical records and electronic anesthesia records. The primary endpoint was an increase in the fibrinogen level after the administration of cryoprecipitate.

**Results:**

Nine patients with no fibrinogen data and four patients with a bleeding volume of less than 2000 mL were excluded; thus, 84 patients (A: *n* = 36, B: *n* = 37, C: *n* = 11) were evaluated. The mean intraoperative blood loss (mL) in groups A, B, and C were 3348 ± 791, 6688 ± 1225, and 14,281 ± 5142, respectively. The fibrinogen levels (mg/dL) before cryoprecipitate administration in groups A, B, and C were 189 ± 94, 113 ± 42, and 83 ± 29, respectively (*p* < 0.05 among the groups). The increase in fibrinogen level (mg/dL) after cryoprecipitate administration in group C was significantly greater than that in group A (84 ± 34 versus 50 ± 36, *p* < 0.01).

**Conclusions:**

The results of this study indicate that the effect of cryoprecipitate on the increase in fibrinogen level was most apparent in patients with excessive intraoperative blood loss ≥ 10,000 mL. In addition, most patients with intraoperative blood loss ≥ 5000 mL had fibrinogen levels < 150 mg/dL which improved to ≥ 150 mg/dL after cryoprecipitate administration in approximately 70% of patients. Therefore, cryoprecipitate administration should be considered for patients with hypofibrinogenemia (≤ 150 mg/dL) experiencing severe bleeding (e.g., ≥ 5000 mL) and rapid administration of cryoprecipitate is necessary to maximize the hemostatic effect, especially when the bleeding volume exceeds 10,000 ml.

## Background

Cryoprecipitate, which is concentrated from fresh frozen plasma (FFP) and contains a large amount of fibrinogen and factor VIII, shows have a hemostatic effect in cases of massive bleeding with hypofibrinogenemia. Recent studies have shown that fibrinogen levels fall below 150 mg/dL during massive intraoperative bleeding [1]. Fibrinogen is essential for clot formation with platelet aggregation; severe hypofibrinogenemia during surgery is assumed to be one of the main causes of dilutional coagulopathy and difficulty in hemostasis [1]. Thus, fibrinogen level is used as a determinant for blood transfusion in cases of massive bleeding may be worthwhile.

Since 2013, cryoprecipitate or fibrinogen concentrate has been recommended as a supplement for fibrinogen supplementation in cases of significant bleeding with functional fibrinogen deficiency or coagulopathy, characterized by fibrinogen levels below 100–150 mg/dL [2]. FFP, which does not contain a high concentration of fibrinogen and requires a long time from dissolution to completion of administration, is inappropriate and insufficient to raise the concentration of blood fibrinogen to the hemostatic level (> 150–200 mg/dL) immediately in patients with persistent bleeding. However, a review on the use of cryoprecipitate revealed that the current guidelines should be limited to the treatment of hypofibrinogenemia in patients with a clinical bleeding tendency [3]. Cryoprecipitate is effective in increasing fibrinogen levels; however, data on its clinical efficacy are limited, and clinical trials conducted in bleeding settings are needed.

In our previous retrospective study, the intraoperative blood loss and transfusion requirements were greater when cryoprecipitate was administered after fibrinogen levels dropped below 100 mg/dL, compared with the corresponding findings for cryoprecipitate administration at fibrinogen levels of 100–150 mg/dL [4]. These findings suggest that intraoperative cryoprecipitate administration should be performed when fibrinogen levels are between 100 and 150 mg/dL. However, if fibrinogen levels cannot be measured at the bedside, the timing of cryoprecipitate administration cannot be conclusively determined. In this study, we retrospectively investigated the effect of cryoprecipitate administration on the increase in fibrinogen levels in patients with excessive intraoperative blood loss.

## Methods

### Study population

This retrospective observational study was conducted with the approval of the Ethics Committee of the University of Miyazaki Hospital (approval number O-1030). A total of 97 patients who received cryoprecipitate intraoperatively between June 2014 and May 2019 were enrolled in this study. The 97 patients enrolled in the present study included 53 patients (54.6%) who were enrolled in a previous retrospective study for the different purpose of examining the effect of cryoprecipitate on intraoperative hemorrhage during surgery [4]. Commercially available fibrinogen concentrates have limited indications in Japan; therefore, we produce and use cryoprecipitate at our institution. We prepared one pack of cryoprecipitate from four units (FFP-LR480) of AB RhD-positive FFP and stored in six packs, as per the method reported previously [5]. Two packs can be used simultaneously during surgery. The decision regarding the administration of cryoprecipitate and the number of packs to be administered was made by the anesthesiologist in charge or the primary surgeon. Fibrinogen values were measured before and after cryoprecipitate administration, and intraoperative blood loss was measured and evaluated. For cardiovascular surgery patients who underwent cardiopulmonary bypass, intraoperative blood loss was calculated including the amount of blood collected by the cell saver system. The patients were divided into three groups based on intraoperative blood loss: group A, blood loss of 2000–5000 mL; group B, blood loss of 5000–10,000 mL; and group C, blood loss > 10,000 mL.

### Data and outcomes

The data for the following parameters were extracted from the electronic medical records and electronic anesthesia records: age, sex, height, weight, American Society of Anesthesiologists physical status (ASA-PS) classification, type of surgery (cardiovascular surgery, obstetrics and gynecologic surgery, and others), surgery time, anesthesia time, and the number of cryoprecipitates used. The primary endpoint was the increase in the fibrinogen level after the administration of cryoprecipitate. We examined the postoperative 30-day mortality as an outcome.

### Statistical analyses

Data are shown as real values, mean ± standard deviation, or median (interquartile range). One-way analysis of variance (ANOVA) and multiple comparisons (Tukey’s HSD test) or Kruskal–Wallis test and multiple comparisons (Steel–Dwass test) were performed for group comparisons; the chi-square test was used for frequency comparisons. Statistical significance was set at a two-tailed *p* < 0.05. The statistical software JMP Pro 15 (SAS Institute, Inc., Cary, NC, USA) for Macintosh was used for statistical analyses.

## Results

**Fig. 1:**
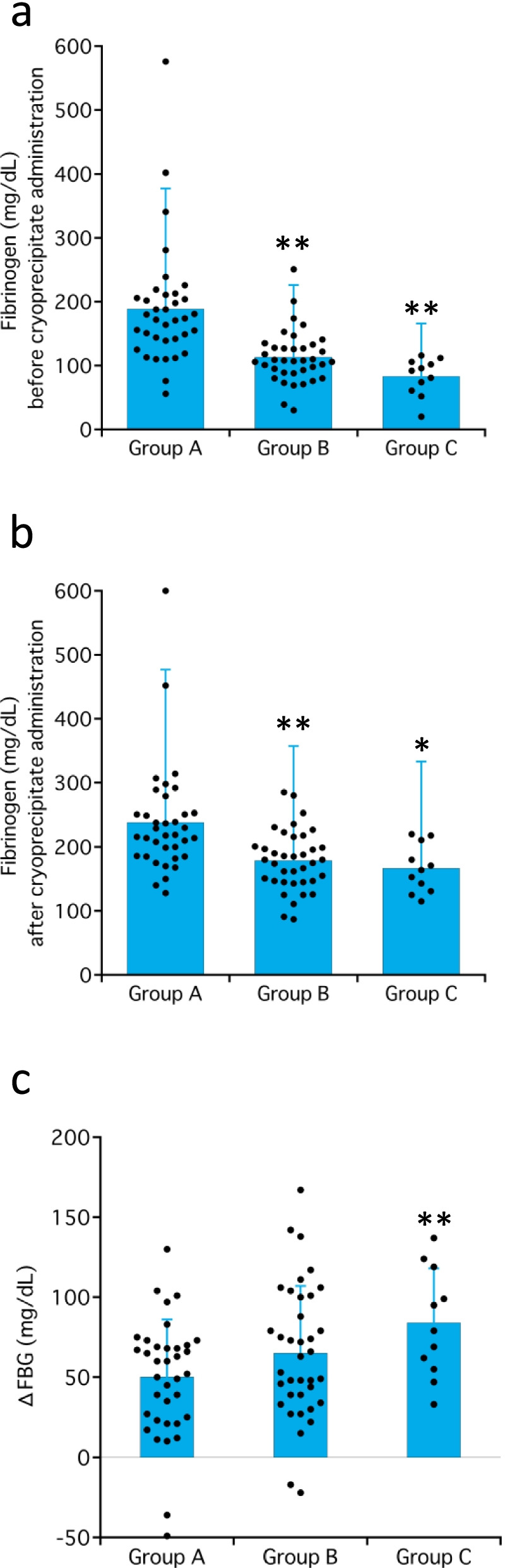
Fibrinogen levels before (**a**) and after (**b**) the administration of cryoprecipitate and their degrees of increase (**c**). The fibrinogen levels were significantly lower in groups B and C than in group A before the administration of cryoprecipitate (**a**). They significantly increased in all groups after the administration of cryoprecipitate (**b**), although they were still significantly lower in groups B and C than in group A after the administration of cryoprecipitate. However, the degree of increase in the fibrinogen level was significantly greater in group C than in group A (**c**). *p < 0.05 and **p < 0.01 vs. Group A

Among the 97 patients, 9 patients for whom fibrinogen was not measured and 4 patients whose intraoperative blood loss < 2000 mL, were excluded. Backgrounds of the remaining 84 patients (*n* = 36, 37, and 11 in groups A, B, and C, respectively) are provided in Table 1. Significant between-group differences were observed in body weight, ASA-PS, operative time, anesthesia time, and the dose of cryoprecipitate. The fibrinogen levels were significantly lower in groups B (113 ± 42 mg/dL) and C (83 ± 29 mg/dL) than in group A (189 ± 94 mg/dL) before cryoprecipitate administration (*p* < 0.0001 for both) (Fig. 1a), which significantly increased after administration of cryoprecipitate in all groups. However, the fibrinogen levels after cryoprecipitate administration remained significantly lower in groups B and C than group A (Fig. 1b). The degree of increase of fibrinogen levels was significantly larger in group C than in group A (Fig. 1c). The three groups differed significantly in partial thromboplastin time (PTT) and activated partial thromboplastin time (APTT) before cryoprecipitate administration. However, no difference was observed after administration (Table 2). Transfusion of red blood cells (RBCs), FFP, and platelet concentrate (PC) increased significantly with intraoperative blood loss. However, there were no significant between-group differences in 30-day mortality rates.

**Table 1:** Patient characteristics

	Group A (*n* = 36)	Group B (*n* = 37)	Group C (*n* = 11)	*p*
Age (years)	62 ± 14	61 ± 18	67 ± 19	0.34
Sex (male/female)	20/16	26/11	6/5	0.37
Height (cm)	157 ± 0	160 ± 8	158 ± 7	0.25
Weight (kg)	55 ± 14	63 ± 12^*^	60 ± 11	0.048
ASA-PS, 1/2/3/1E/2E/3E/4E	0/2^†^/18/0/2/13^†^/1	0/10/16/0/1/6/4	0/3/6/1^††^/0/0/1	0.02
Surgery				
Cardiovascular/obstetric/other	33/2/1	30/4/3	8/2/1	0.55
Operation time (min)	426 ± 148	533 ± 212	660 ± 266^**^	0.002
Anesthesia time (min)	527 ± 174	643 ± 254	774 ± 307^**^	0.006
Intraoperative blood loss (ml)	3,348 ± 791	6,688 ± 1,225^**^	14,281 ± 5,142^**^	< 0.001
Cryoprecipitate, 2/4/6 packs	33/3/0	34/2/1	7^††^/4^††^/0	0.04

*ASA-PS* American Society of Anesthesiologists physical status classification

*p < 0.05 vs. Group A, **p < 0.01 vs. Group A, ^†^*p* < 0.05 vs. expected value, ^††^*p* < 0.01 vs. expected value

**Table 2:** Coagulation capacity before and after administration of cryoprecipitate, intraoperative blood transfusion volume, and outcome

	Group A (*n* = 36)	Group B (*n* = 37)	Group C (*n* = 11)	*p*
Pre-PTT (s)	16.0 (14.2–17.9)	17.6 (16.3–19.2) ^**^	19.7 (17.4–24.0) ^**^	0.003
Post-PTT (s)	14.2 (13.6–14.8)	14.4 (13.7–16.0)	14.1 (13.5–15.5)	0.37
Pre-APTT (s)	40.8 (37.5–49.9)	51.2 (43.5–109.5) ^**^	85.2 (79.5–180.0) ^**^	0.001
Post-APTT (s)	36.1 (33.3–38.1)	38.5 (32.3–45.4)	35.3 (32.0–60.4)	0.34
Transfusion (ml)				
RBC	1540 ± 420	2240 ± 1400^*^	4620 ± 1820^****, ††††^	< 0.0001
FFP	960 ± 600	1800 ± 1200^***^	4680 ± 2400^****, †††^	< 0.0001
PC	380 ± 180	480 ± 320^**^	520 ± 180^**^	0.001
30-day mortality, *n* (%)	3 (8)	5 (14)	0 (0)	0.39

*PTT* partial thromboplastin time, *APTT* activated partial thromboplastin time, *RBC* red blood cell, *FFP* fresh frozen plasma, *PC* platelet concentrate

*p < 0.05 vs. Group A, **p < 0.01 vs. Group A, ***p < 0.001 vs. Group A, ****p < 0.0001 vs. Group A, ^†^*p* < 0.05 vs. Group B, ^†††^*p* < 0.001 vs. Group B, ^††††^*p* < 0.0001 vs. Group B

## Discussion

Continuous intraoperative bleeding causes a reduction in coagulation factor levels. Pathological conditions such as hypothermia and acidosis complicate coagulation reactions based on enzyme-substrate reactions. Coagulation factor levels decrease as the amount of bleeding increases, leading to a vicious cycle of further bleeding and difficulty in stopping the bleeding [6]. Our previous study revealed a negative correlation between the fibrinogen levels and bleeding volume before cryoprecipitate administration, and the amount of blood loss and transfusion tended to increase when the fibrinogen levels were < 100 mg/dL [4]. In the present study, the operative time was significantly prolonged in the group with higher blood loss, which was expected to lead to further blood loss and difficulty in stopping the bleeding. In a systematic review comparing the fibrinogen concentrate with FFP, 5 out of 91 eligible studies reported the outcome of fibrinogen concentrate versus a comparator [7]. The evidence was consistently positive (70% of all outcomes), with no negative effects reported (0% of all outcomes). Fibrinogen concentrate was compared directly with FFP in three high-quality studies and was found to be superior for > 50% of outcomes in terms of reducing blood loss, allogeneic transfusion requirements, length of intensive care unit and hospital stay, and increasing the plasma fibrinogen levels. However, it was concluded that the trigger value for fibrinogen administration in acquired fibrinogen deficiency, a common coagulation factor deficiency condition associated with surgical bleeding, remains unclear. Many guidelines and mass transfusion protocols recommend the administration of cryoprecipitate when the plasma fibrinogen levels are below 100–150 mg/dL; however, this threshold is not based on established clinical evidence [2]. In addition to the increased consumption of clotting factors in the setting of persistent bleeding, the difficulty in obtaining a timely measurement of the fibrinogen levels has led to the argument that fibrinogen levels should not be used as a guide for the management of bleeding [8]. Besides measuring fibrinogen levels, viscoelastic devices such as thromboelastography (TEG) and rotational thromboelastometry (ROTEM) are useful as they can provide real-time data regarding severe bleeding, especially in relation to cardiovascular surgery; the measurement of blood viscosity by viscoelastic devices in the operating room (point-of-care monitoring) is ideal. This strategy has the potential to reduce the risk of complications such as transfusion-associated circulatory overload (TACO), transfusion-related acute lung injury (TRALI), and thromboembolic adverse events [9]. Our previous study concluded that plasma fibrinogen levels should be maintained between 100 and 150 mg/dL and should be > 100 mg/dL to prevent clotting factor deficiency [4]. To prevent hypofibrinogenemia, plasma fibrinogen levels should be maintained > 100–150 mg/dL and not < 100 mg/dL. Kikura et al. reported that a fibrinogen level < 130 mg/dL is the cutoff value for fibrin polymerization (FIBTEM A10) measured by ROTEM as < 6 mm, and that postoperative blood loss and transfusion volume significantly increased when FIBTEM A10 was < 6 mm [10]. They also found that the effect of fibrinogen concentrate on reducing blood loss and transfusion volume was clear in patients with FIBTEM A10 < 6 mm at the time of weaning from cardiopulmonary bypass, while in patients with FIBTEM A10 > 6 mm, hemostatic capacity was high and FFP was sufficient [11]. In addition to these viscoelastic devices, new techniques for rapid measurement and estimation of fibrinogen levels have recently been developed in Japan [12, 13]. Although there are limitations in the interpretation and these rapid measurements of fibrinogen levels, it is hoped that rapid measurement of fibrinogen levels will become more widespread in the future, as fibrinogen levels themselves remain an important guideline for evaluating patients with bleeding [14].

In the present study, group C, who experienced significant bleeding and fibrinogen concentrations of approximately < 100 mg/dL before cryoprecipitate administration, demonstrated significantly greater fibrinogen recovery than group A, who experienced relatively little bleeding and maintained the fibrinogen levels. However, the dose of cryoprecipitate administered in relation to the amount of bleeding must be considered, since the percentage of the four packs of cryoprecipitate used in group C was significantly higher. In addition, a high initial fibrinogen concentration at the start of fibrinogen supplementation limits further increments in fibrinogen concentration [15]. These findings suggest that the timing of cryoprecipitate administration is important to maximize the effect of cryoprecipitate, taking into account the amount of bleeding and the decrease in fibrinogen level due to bleeding.

In situations where viscoelastic devices or rapid fibrinogen measurement devices have not been introduced, the decision to administer cryoprecipitate must be made comprehensively based on the amount of blood loss, the rate of blood loss, the progress of the surgery, and vital signs. Our findings might help determine the timing of cryoprecipitate administration using the amount of intraoperative blood loss as an indicator. In our study, most patients showed fibrinogen levels > 100 mg/dL and approximately 60% of the patients showed fibrinogen levels > 150 mg/dL in group A, whereas half of the cases in group B and 70% of those in group C had fibrinogen levels < 100 mg/dL. Since most patients in group B had fibrinogen levels < 150 mg/dL which improved to > 150 mg/dL after cryoprecipitate administration in approximately 70% of patients, this observation might support the hypothesis that cryoprecipitate administration should be considered when bleeding exceeds 5000 ml and rapid cryoprecipitate administration is necessary for hemostasis when bleeding is > 10,000 mL.

The present study has several limitations. The surgeries were classified according to the amount of blood loss, and the surgical site was not considered. The background may be different for obstetric hemorrhage, wherein coagulation factors are lost early, and cardiovascular surgery, wherein the consumption of coagulation factors by artificial heart-lung, hemodilution, hypothermia, and effects on platelet aggregation function are taken into account. In addition, a multicenter prospective study is desirable in the future, since this was a single-center retrospective study.

## Conclusion

The results of this study indicate that the effect of cryoprecipitate on the increase in fibrinogen level was most apparent in patients with excessive intraoperative blood loss ≥ 10,000 mL. In addition, most of the patients with intraoperative blood loss ≥ 5000 mL had fibrinogen levels < 150 mg/dL which improved to ≥ 150 mg/dL after cryoprecipitate administration in approximately 70% of patients. Therefore, cryoprecipitate administration should be considered for patients with hypofibrinogenemia (≤ 150 mg/dL) experiencing severe bleeding (e.g., ≥ 5000 mL when the preoperative fibrinogen level is within the normal range) and rapid administration of cryoprecipitate is necessary to maximize the hemostatic effect, especially when the bleeding volume exceeds 10,000 ml.

## Data Availability

The datasets used and/or analyzed during the current study are available from the corresponding author upon reasonable request.

## Abbreviations

FFP: Fresh frozen plasma
FFP-LR: Fresh frozen plasma-leukocytes reduced
ASA-PS: American Society of Anesthesiologists physical status; HSD: honestly significant difference
IQR: Interquartile range
ANOVA: Analysis of variance
PTT: Partial thromboplastin time
APTT: Activated partial thromboplastin time
RBCs: Red blood cells
PC: Platelet concentrate
TEG: Thromboelastography
TACO: Transfusion-associated circulatory overload
TRALI: Transfusion-related acute lung injury
